# Constructe a novel 5 hypoxia genes signature for cervical cancer

**DOI:** 10.1186/s12935-021-02050-3

**Published:** 2021-07-03

**Authors:** Yang Yang, Yaling Li, Ruiqun Qi, Lan Zhang

**Affiliations:** Department of Dermatology, The First Hospital of China Medical University and National Joint Engineering Research Center for Theranostics of Immunological Skin Diseases, The First Hospital of China Medical University and Key Laboratory of Immunodermatology, Ministry of Health and Ministry of Education, Shenyang, 110001 China

**Keywords:** Hypoxia, Cervical cancer, Prognosis, Bioinformatics

## Abstract

**Background:**

Hypoxia, which affects the development, metastasis and prognosis of cancer, represents a key feature of cancer. This study describe a hypoxia risk factor model, with predicting the prognosis of cervical cancer.

**Methods:**

Based on hypoxia pathway related genes, we divided cervical cancer samples into high and low expression groups. A cox analysis was then performed. Genes from these cervical cancer samples showing a significant impact on OS were selected for cluster analysis to obtain two subtypes. The TPM dataset of TCGA was divided into training and validation sets. For the training set, a lasso analysis was conducted as based on cox analysis of meaningful genes and a risk factor model was constructed. The constructed model was verified in internal and external data sets. Finally, RT-PCR, immunohistochemistry were used to detect the expression of relative genes or proteins and functional assays were used to evaluate the biological function of signature genes.

**Results:**

Two molecular subtypes were obtained, Cluster2 vs Cluster1.These subtypes were obtained by clustering with a total of 149 DEGs (Differential expressed genes) being in line with this standard, of which 27 were up-regulated and 122 were down-regulated. The five genes with lambda = 0.0571 were selected to construct the model, the RiskScore = AK4*0.042 + HK2*0.021 + P4HA1*0.22 + TGFBI*0.1 + VEGFA*0.077. Further, in order to verify the signature, we used TCGA-test and GSE44001 chip datasets to test, and finally got a good risk prediction effect in those datasets. Moreover, the result of RT-PCR and immunohistochemistry demonstrated that *AK4, HK2, P4HA1, TGFBI* and *VEGFA* were all highly expressed in these cervical cancer tissue samples. The functional study shown that expression of *AK4, HK2, P4HA1, TGFBI* and *VEGFA* can regulate the proliferation, migration, and invasion ability of cervical cancer cells in vitro.

**Conclusions:**

In summary, we developed a 5-gene signature prognostic hierarchical system based on the hypoxic pathway of cervical cancer, which is independent of clinical characteristics. And also conducted experimental verifications on these signature gene. Therefore, we propose that use of this classifier as a molecular diagnostic test can provide an effective means for evaluating the prognostic risk of cervical cancer patients, and provide potential targets for the treatment of cervical cancer patients.

**Supplementary Information:**

The online version contains supplementary material available at 10.1186/s12935-021-02050-3.

## Background

The most recent survey results indicate that cervical cancer ranks fourth in morbidity and mortality among women [1]. The findings that the incidence of this cancer varies among different countries and regions reveals that considerable geographic diversity exist. In addition, risk factors related to social and economic conditions are present within specific regions [2]. Persistent human papillomavirus (HPV) infection is a major cause of cervical cancer, with poor prognosis in developing countries [3, 4]. And the vaccine only targeted women before infection, will not help women who have been infected with HPV. CT and MRI are considered to be the most effective tools for use in the clinical evaluation of invasive cervical cancer [5], while PET-CT has become increasingly more common in preoperative examinations and diagnoses of metastatic cervical cancer. However, difficulties in detecting lymph node metastases and limited spatial resolutions diminish PET-CT’s ability for detecting small lesions or early screening and diagnosis of primary lesions [6–8]. Cervical cancer is aggressive and often only identified in advanced stages. For women with metastatic or recurrent diseases, the overall prognosis remains poor [9]. As the prognosis of cervical cancer varies markedly as a function of patient’s genetic heterogeneity, it is clear that the identification of effective genetic biomarkers would significantly enhance the capacity to predict the prognosis of this condition [10–12].

With the maturity of high-throughput sequencing technology, large-scale omics data generation is allowed [13, 14]. Signatures of these hypoxia-related genes may explain cancer etiology and could hold diagnostic and prognostic value. However, cervical cancer has no hypoxia-associated prognostic signature has been established.

Hypoxia is a common cause of many malignant tumors and plays an important role in promoting the growth and metastasis of various tumors including cervical cancer [15, 16]. Hypoxia activates several pathways leading to tumor epithelial-mesenchymal transition (EMT). EMT is a basic process of cancer invasion and involves different regulatory mechanisms depending on the types and subtypes of malignant tumors. Typically, cells undergoing this transformation lose many epithelial markers and ZO-1 expression, while mesenchymal markers are increased [17]. In addition, under hypoxic conditions, cancer cells exhibit adaptive metabolic changes, including the conversion of glucose to lactic acid and increased glucose uptake by promoting glucose transporters (GLUTs), known as the Warburg effect [18]. Results from a number of studies have shown that hypoxia is associated with the prognosis of many tumors [19–22]. Since hypoxia genes are closely related to the occurrence and development of cervical cancer, we attempt to develop a predictive model on the basis of hypoxic-related differential genes to provide accurate clinical guidance to cervical cancer patients. Although there have been some studies directed at developing prognostic models as based on gene signatures, few studies have focused on hypoxia-related genes.

In our study, hypoxia genes were considered to be closely related to the progression of cervical cancer. By analyzing the relationship between the expression of hypoxia-related genes and cervical cancer patients, we established a prognostic prediction model based on hypoxia genes, and evaluated the stability of the model through internal and external validation sets, which proved that the model is effective and possesses good predictive ability. This has not been reported in previous studies. In the future diagnosis and treatment of cervical cancer, genetic diagnosis and treatment will become effective means. By detection of the expression of model genes in cervical cancer tissues, and the conversion of model genes into risk scores, it is used to predict the prognosis of patients and has a clinical application value.

## Materials and method

### Data download and preprocessing

The gene expression profile and clinical follow-up information on cervical cancer patients were downloaded from TCGA and Gene Expression Omnibus (GEO) data (GSE44001). For processing of the RNA-Seq data of TCGA, while samples lacking clinical follow-up information were eliminated. Processing of the GEO dataset was performed in the following sequence, consisting of elimination of samples without clinical follow-up information survival time and status, transformation of the probe into a Gene Symbol, removal of probes corresponding to multiple genes and retention of maximal expression values with multiple Gene Symbols. For the hypoxia gene dataset, genes of the Hypoxia-related pathway (HALLMARK_HYPOXIA) were downloaded from MSigDB and 200 Hypoxia-related genes were sorted out.

### Cluster analysis of TCGA cervical cancer

Initially, 200 genes related to the hypoxia pathway were downloaded from MSigDB(http://www.gsea-msigdb.org/gsea/msigdb/index.jsp) for analysis. These samples were then divided into high and low expression groups according to the median of gene expression in the TCGA-TPM data of cervical cancer. A cox analysis, as based on overall survival (OS), was performed and genes with a p < 0.05 were screened. A forest map was then drawn using the forestplot package to illustrate results of the cox analysis. A Cluster analysis was performed on TCGA-TPM data of cervical cancer samples with genes selected that affected the OS. This Cluster analysis employed the R package ConsensusClusterPlus, with the parameters of the clustering algorithm being k-means and the distance parameter euclidean. According to the CDF (Cummulative distribution function) and Delta Area, 2 represents the optimal K value. The OS-based survival curve of samples in the Cluster was then drawn.

### Identification of differentially expressed genes (DEGs) and functional enrichment analysis

The limma package was used to analyze the two subtypes, as obtained from clustering. The principle for screening differential genes was abs (logFC) > 1.2 and an adj. of p < 0.05.Genes showing differential expressions were identified to construct a volcanic map with ggplot2 package. With use of the clusterprofiler package, GO (Gene Ontology) and KEGG (Kyoto Encyclopedia of Genes and Genomes) pathway analyses were performed on DEGs.

### Grouping TCGA cervical cancer dataset

The 257 samples in the TCGA data set were divided into a training and test set, according to the proportion of training set:test set = 7:3. Finally, training and test set samples were displayed, and the chi-square test was used to verify the rational for the grouping.

### Construction of prognostic model based on genes related to the hypoxia pathway

A cox analysis was initially performed on the training set data based on genes related to the hypoxia pathway. The samples were divided into high and low expression groups based on the median of gene expression and the effects of high and low expression on prognosis were analyzed, with genes affecting OS being screened (p < 0.05). Then, as based on the above hypoxia-related pathway genes demonstrating an impact on OS, the R software package glmnet, was used to perform a lasso cox regression analysis on data of the test set samples. The trajectory of independent variables reveals that with a gradual decrease in lambda, the number of independent variable coefficients tending to 0 increases gradually. A tenfold cross validation was used to construct the model and analyze confidence intervals under each lambda. With a lambda = 0.0571, the model is optimally effective, therefore genes at this point were used to construct the model. Survival curves were then drawn for the training set of the genes used for modeling.

### Construction, evaluation and verification of the prognostic model

Within the training set, RiskScore of each sample were calculated according to expression levels of the sample and the RiskScore distribution was drawn for the sample. Next, the ROC analysis for the prognostic classification of RiskScore was performed using the R package, pROC. As based on the calculated median of RiskScore, samples were divided into high and low risk groups and survival curves were plotted. As a means to assess the robustness of this model, the same model and coefficient as the training set was used for the internal validation set and all data sets of TCGA. The predictive effect of RiskScore between high and low risk groups were evaluated. Next, the same coefficient as the training set for the external validation data set GSE44001 were used. Again, the predictive effect of RiskScore on the prognosis between the high and low risk groups were performed.

### Risk assessment of clinical subtypes

A boxplot was then used to show the distribution of RiskScore in Grade3/4 and Grade1/2samples. Clinical subtypes such as Grade (1/2), Grade (3/4), Stages (I/II), Stages (III/IV), Age (> 65) and Age (≤ 65) were grouped as based on the expression of Risk score to evaluate the prognosis of high and low risk groups.

### Univariate and multivariate cox analyses

Univariate and multivariate COX regressions were used to analyze relations among risk scores, other variables and clinical prognosis of patients.

### RiskScore and clinical features for construction of the nomogram

Based on results of the univariate and multivariate analyses a nomogram model was constructed with the clinical features Age, Stage and RiskScore, and as a means to generate a calibration curve to evaluate the prediction accuracy of the model. In addition, the DCA (Decision Curve Analysis) was used to assess the reliability of the model.

### Tissue samples

Cervical cancer samples and adjacent non-tumor tissues were collected from 10 patients (all > 16 years of age), immediately placed in liquid nitrogen and preserved at – 80 °C. Cervical cancer samples including 7 cervical squamous cell carcinomas and 3 cervical adenocarcinoma. None of the patients received any preoperative anti-tumor therapies. All patients and their families were fully informed as to the purpose of the study and informed consent was obtained from all participants. This study was approved by the Ethics Committee of the First Hospital of China Medical University (2016-207-4).

### Cell lines and culture conditions

Cervical cancer cells, Hela, were purchased from the Chinese Academy of Medical Sciences and the CAMS & PUMC Medical College (Beijing, China). Cell lines were cultured in 1640 medium supplemented with 10% fetal bovine serum and 100 units/ml penicillin at 37 °C in a humidified 5% CO_2_ incubator.

### RNA isolation and RT-PCR analysis

RNA extraction from tissues was performed using the TRIzol reagent (Invitrogen, Carlsbad, CA, USA). RNA was reverse-transcribed into cDNA with use of the QuantiTect Reverse Transcription Kit (QIAGEN, Valencia, CA, USA). Real-time PCR analyses were quantified by SYBR-Green (Takara, Otsu, Shiga, Japan) and levels were normalized to GAPDH levels. Sequences of upstream and downstream primers are contained in Additional file 11: S6.xls.

### Immunohistochemistry

Cervical cancer samples were fixed in 10% formalin, embedded in paraffin and processed as 5-µm sequential sections. Samples were dewaxed with ethanol and blocked to inhibit endogenous peroxidase activity. They were then heated in a microwave to retrieve antigens, cooled to room temperature and then blocked by incubation in goat serum for 30 min at 37◦C. Samples were incubated overnight at 4 °C with rabbit anti-AK4 (PA5-60,104), anti-HK2 (PA5-83,021), anti-P4HA1 (PA5-18,669), anti-TGFBI (PA5-82,358), and anti-VEGFA (PA5-85,171) (Thermo Fisher Scientific MA, USA; 1:1, 200), followed by incubation with horseradish peroxidase-coupled goat anti-rabbit secondary antibody at 37 °C for 30 min and then stained using 3,3′-diaminobenzidine. Cell nuclei were stained blue with use of hematoxylin. Sections were then dehydrated, cleared with xylene, and mounted. *AK4, HK2, P4HA1, TGFBI* and *VEGFA* expressions were determined with use of IHC (Immunohistochemistry) using a streptavidin peroxidase method, with adjacent tissues as controls. The experimental procedure was performed according to the manufacturers’ instructions. TheImage-ProPlus 6.0 Software (MediaCybernetics, USA) was used to analyze protein expressions and perform statistics on the results obtained from immunohistochemistry.

### RNA interference

SiRNAs against AK4 (NO. HSS164266), HK2 (NO. HSS179238), P4HA1 (NO. HSS107536), TGFBI (NO.HSS186313), VEGFA (NO.HSS111274) and negative control siRNA sequences from Thermo Fisher Scientific (MA, USA). The transfection was performed using Lipofectamine 3000 reagent (Invitrogen, Shanghai, China) according to the manufacturers’ instructions. Cells were harvested for cytological analysis at 48 h post-transduction.

### Cell migration and invasion assays

Cells were harvested, resuspended in serum-free media and placed in the upper chamber of a Transwell membrane filter (Corning, NY, USA) for migration assays or in the upper chamber of a Transwell membrane filter coated with Matrigel (Corning) for invasion assays. Culture medium with 10% FBS was added to the lower compartment of the chamber to serve as a chemoattractant. After 24 h of incubation, cells were stained with methanol and 0.1% crystal violet, imaged and counted using an Olympus microscope (Tokyo, Japan).

### Generation of single-cell-derived cellular clones

Cell densites were adjusted to 5 × 10^3^/ml and cell suspensions were contained in 100 µl per well in the 6-well plates with 2 ml of DMEM supplemented with 10% FBS and 100 U/ml penicillin. Single-cell-derived clones were grown for 7 days. Cultures were pre-cooled three times with PBS, the methanol was fixed for 15 min, crystal violet dye was applied for 20 min and the water was rinsed and air dried. The number of visible clones was visually counted and a clone formation rate was calculated: clone formation rate (%) = (clone number/number of inoculated cells) × 100%. This procedure was replicated three times.

### Statistical analysis

All data were analyzed using the SPSS 21.0 statistical software program (IBM Corporation, Armonk, NY, USA). Graphs were generated with GraphPad Prism 8.0 Software (GraphPad Software, Inc., San Diego, CA). Student’s t-tests were used. For t-tests, a two-tailed *p* < 0.05 was required for results to be considered as statistically significant.

## Results

### Sample information statistics

**Fig. 1:**
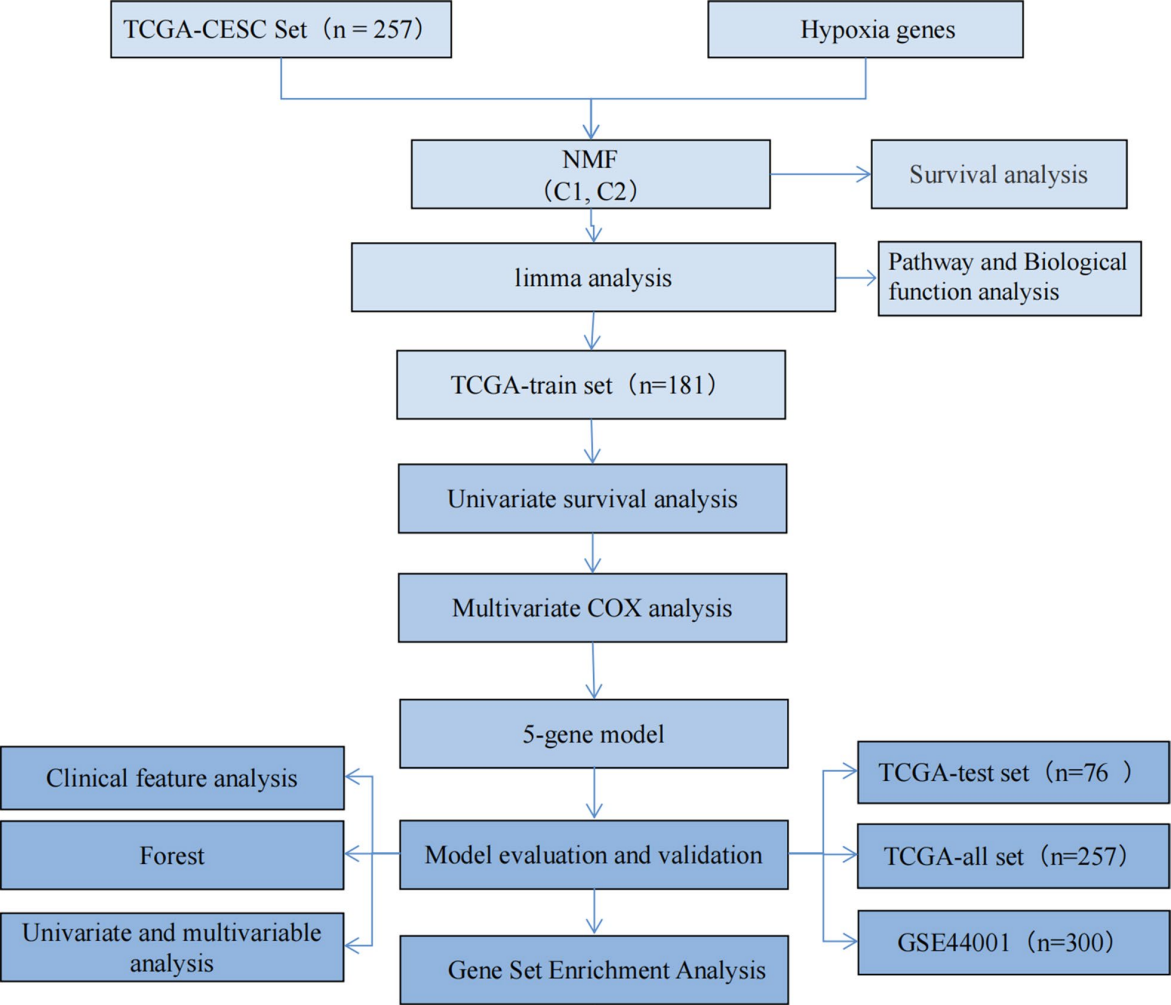
Flowchart of development and validation of a hypoxia genes-based prognostic signature for cervical cancer

The gene expression profile and clinical follow-up information on cervical cancer patients were downloaded from TCGA and GEO data (GSE44001). A total of 557 samples were obtained, including 257 TCGA and 300 GSE44001 samples. The steps to establish the model of prognostic characteristics are as Fig. 1. The preprocessed cervical cancer dataset is contained in Table 1.

**Table 1:** Clinical information of cervical cancer cohort

Clinical features	TCGA-CESC	GSE44001
OS		DFS
0	188	262
1	69	38
Stage		
Stage I	138	
Stage II	58	
Stage III	36	
Stage IV	19	
Grade		
G1	16	
G2	116	
G3	99	
G4	1	
Age		
≤ 65	229	
> 65	28	

**Fig. 2:**
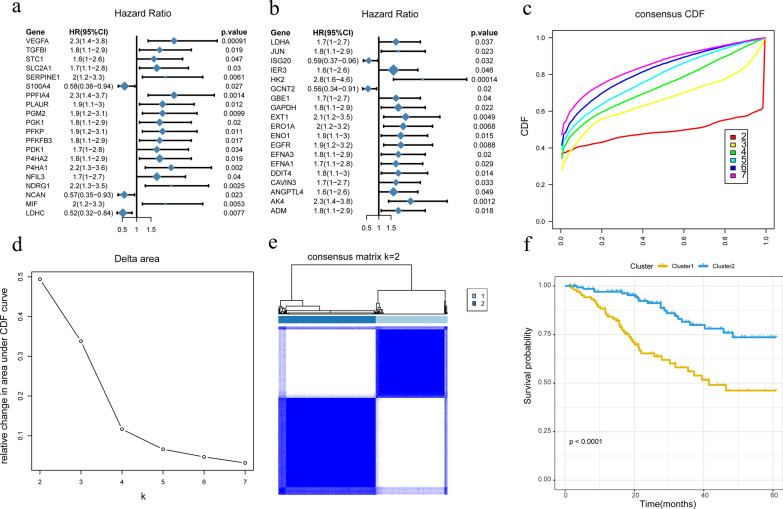
Cox analysis of hypoxia pathway genes. **a** Results 1. **b** Results 2. **c** Cumulative distribution map of clustering consistency. **d** Clustering Delta Area map. **e** Display of clustering results of hypoxia pathway genes. f OS prognosis survival curves of different Clusters

For describing a hypoxia cervical cancer risk factor model, 200 genes related to the hypoxia pathway downloaded from MSigDB for analysis. The TCGA-TPM data of cervical cancer samples were divided into high and low expression groups according to the median of gene expression and a cox analysis as based on OS (the cox analysis results of all hypoxia pathway-related genes are shown in Additional file 1: S1.csv) was conducted. Genes with a p < 0.05 were screened and a forest plot package was used to draw the forest map for display of the cox analysis results (Fig. 2a, b). Among these genes, S100A4, NCAN, LDHC, ISG20 and GCNT2 (HR < 1, p < 0.05) were identified. VEGFA, TGFBI, STC1, SLC2A1, SERPINE1, PPFIA4, PLAUR, PGM2, PGK1, PFKP, PFKFB3, PDK1, P4HA2, P4HA1, NFIL3, NDRG1, MIF, LDHA, JUN, IER3, HK2, GBE1, GAPDH, EXT1, ERO1A, ENO1, EGFR, EFNA3, EFNA1, DDIT4, CAVIN3, ANGPTL4, AK4 and ADM (HR > 1, p < 0.05) were found to have characteristics of high expression and poor prognosis. The TCGA-TPM data of cervical cancer samples were analyzed with the above genes that affected the OS. According to the CDF and Delta Area, a value of 2 indicates the optimal K (Fig. 2c, d). Results from plots of OS-based survival curves of the samples in the two Clusters revealed that the capacity for prognosis from samples in Cluster2 was significantly increased over that in Cluster1 (Fig. 2e, p < 0.0001).

### Differential genes analysis of two molecular subtypes

The limma package was used for the difference analysis of the two subtypes, Cluster2 vs Cluster1. These subtypes were obtained by clustering with a total of 149 DEGs being in line with this standard, of which 27 were up-regulated and 122 were down-regulated. Differentially expressed genes were displayed with use of ggplot2 package (Additional file 2: Fig. S1a, Additional file 3: S2.csv). Heat map of was drawn for the display of these results (Additional file 2: Fig. S1b).

### KEGG pathway and Go enrichment analysis

Furthermore, we performed KEGG pathway and GO functional enrichment analyses on differentially expressed genes. With use of the clusterprofiler package, 27 up-regulated and 122 down-regulated genes were analyzed to determine their biological functions and pathways. A Barplot was used to display the eight enriched KEGG pathway (Additional file 4: Fig. S2a), Biological Process (Additional file 4: Fig. S2b) and Molecular Function (Additional file 4: Fig. S2c), with a detailed analysis of these results presented in Additional file 5: S3.csv and Additional file 6: S4.csv. The KEGG pathway includes ECM-receptor interaction, focal adhesion, IL-17 signaling pathway, PI3K-Akt signaling pathway and the TGF-beta signaling pathway. The Biological Process terms includes extracellular matrix organization, epithelial cell proliferation, cell–matrix adhesion, cell adhesion mediated by integrin, positive regulation of epithelial to mesenchymal transition, regulation of epithelial to mesenchymal transition. The Molecular Function terms includes extracellular matrix structural constituent, receptor ligand activity, cytokine activity, extracellular matrix binding, epidermal growth factor receptor binding, growth factor binding, chemokine activity and interleukin-1 receptor binding.

### Grouping TCGA cervical cancer dataset

For Construction of a prognostic risk model, the 257 samples of the TCGA cervical data set were divided into training and test set according to the proportion of training set: test set = 7:3. The final training and test set samples are displayed and the chi-square test was used to indicate that the grouping was unbiased (Table 2, p > 0.05).

**Table 2:** Grouping information of training set and validation set

Clinical features	TCGA-test	TCGA-train	p
OS			
0	53	135	0.518
1	23	46	
Grade			
1/2	34	98	0.215
3/4	42	83	
Stage			
I/II	61	141	0.799
III/IV	15	40	
Age			
> 65	9	19	0.923
≤ 65	67	162	

### Generation of the prognostic model as based on genes related to the hypoxia pathway

**Fig. 3:**
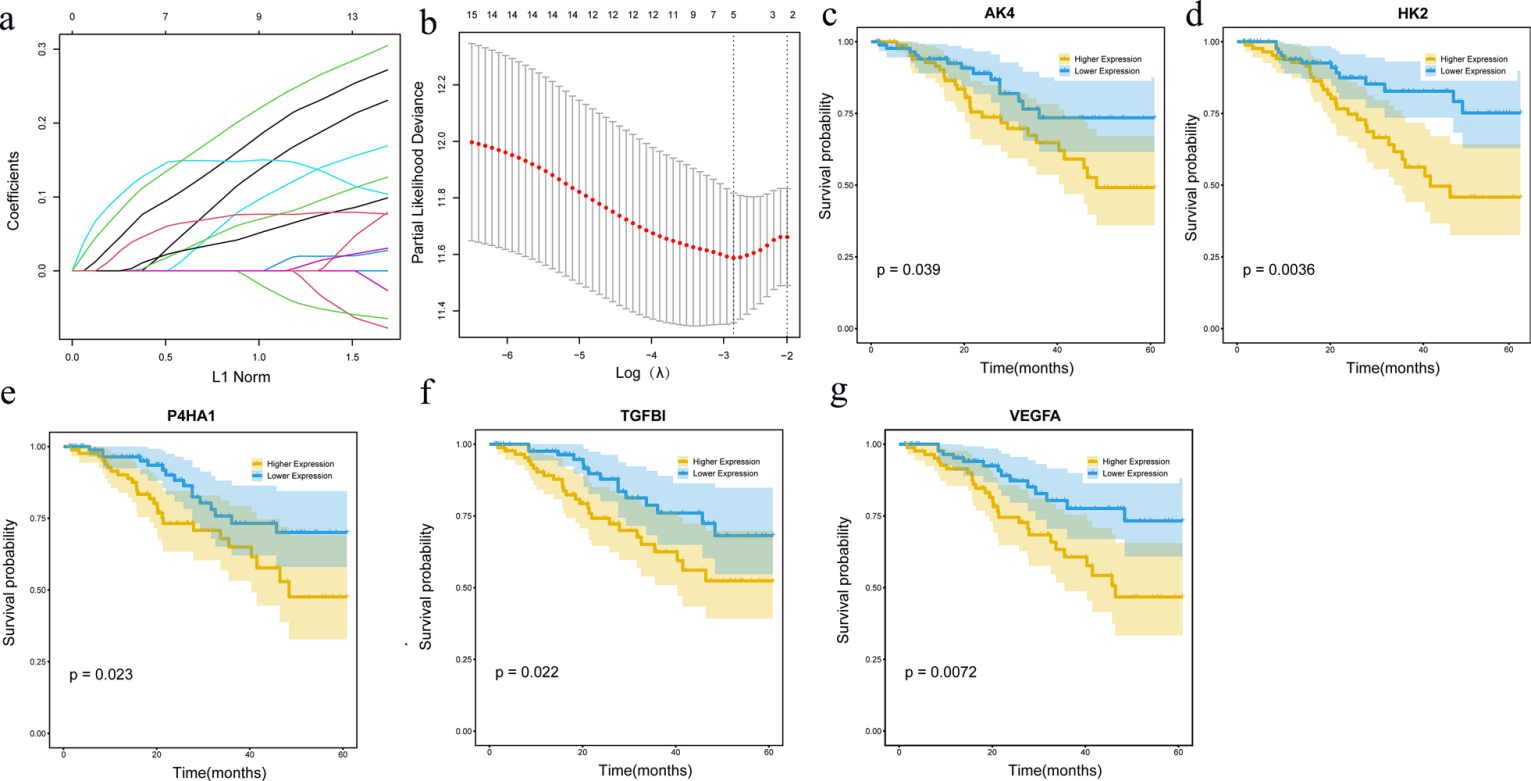
**a** Changes in trajectory for each independent variable with the horizontal axis representing the L1 norm and vertical axis the coefficient of the independent variable. **b** Confidence intervals under each lambda. **c-g** Survival analysis curves for the five prognostic model genes

Training set datas were used for further analyse, Univariate Cox analysis was performed on differential genes among molecular subtypes, and Lasso regression was used to reduce the gene numbers in the risk model. As the lambda value decreases, the number of independent variable coefficients trending toward 0 increases (Fig. 3a). As indicated in Fig. 3b, the model was optimal when lambda = 0.0571. Therefore, the five genes with lambda = 0.0571 were selected to construct the model, the RiskScore = AK4*0.042 + HK2*0.021 + P4HA1*0.22 + TGFBI*0.1 + VEGFA*0.077.

For the five genes used in the model (Fig. 3c–g), survival curves were drawn in the training set. The results show that all 5 genes can significantly distinguish the prognosis of high and low groups.

### Construction, evaluation and validation of the risk model

**Fig. 4:**
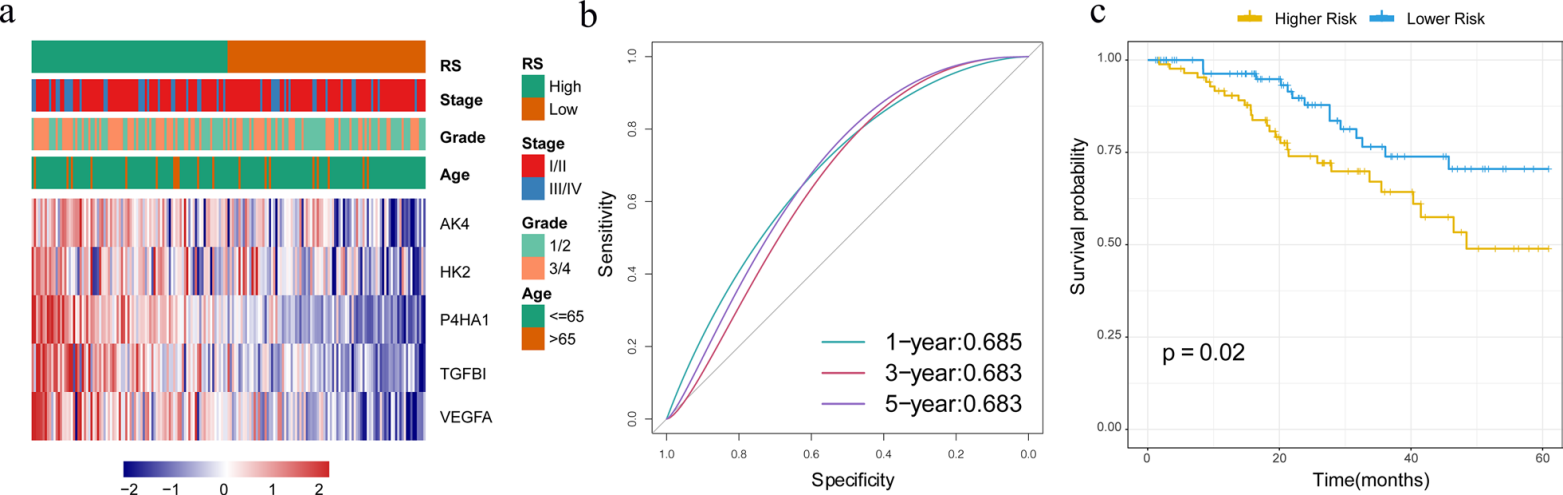
**a** Expression and clinical features of the 5 prognostic genes in the high and low risk groups of the training set. **b** ROC curves and AUC of RiskScore classifications. **c** KM survival curve distribution of the RiskScore in the training set

To determine the robustness of the model, we used the TCGA training set, test set, all TCGA datasets and GSE44001 datasets to calculate RiskScore and drew the RiskScore distribution. In the training set, risk scores of each sample were calculated according to the expression level of the sample with an illustration of the RiskScore distribution of samples shown in Fig. 4a. As is evident from this figure, High expression of *AK4, HK2, P4HA1, TGFBI* and *VEGFA* were associated with a high risk, which indicates a risk factor. ROC analysis of riskScore prognostic classification using the R software package, pROC, revealed that the AUC (Area Under the Curve) of the model was greater than 0.68 at 1, 3 and 5 years (Fig. 4b). According to the median of RiskScore, cervical cancer samples were divided into High RiskScore group and Low RiskScore group, and the survival curve was plotted by KM analysis. It can be seen that the prognosis of Low RiskScore risk group was significantly better than that of High RiskScore group (Fig. 4c).

**Fig. 5:**
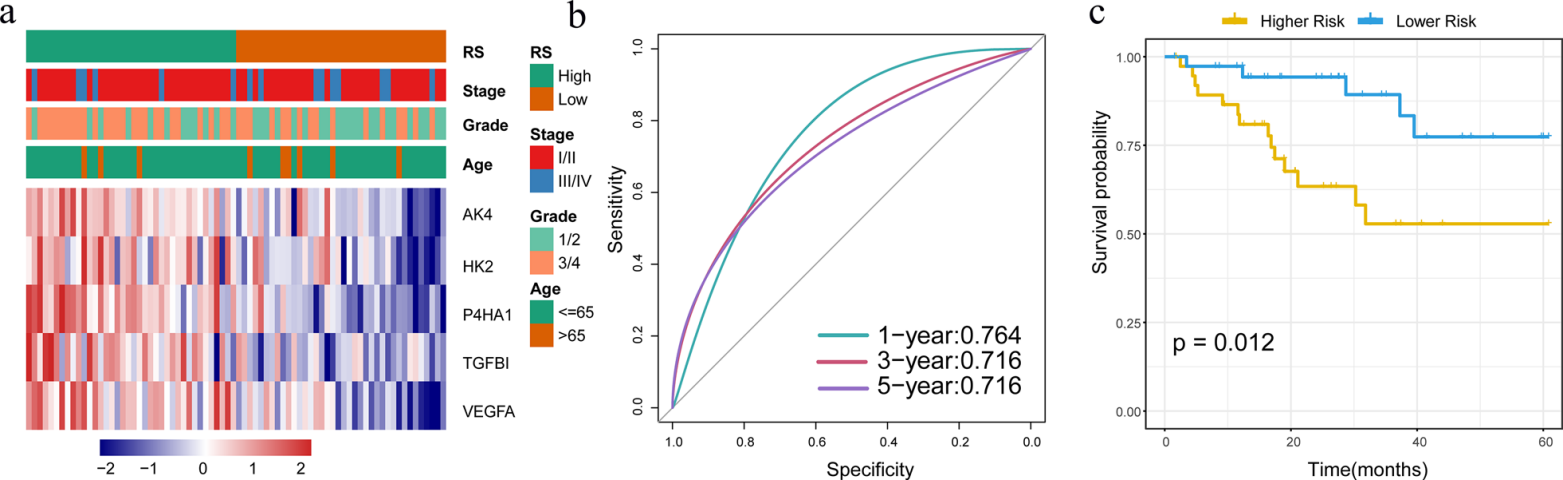
**a** Verification of expressions and clinical characteristics of the five prognostic genes in the high and low risk groups. **b** ROC curves and AUC of RiskScore classifications. **c** RiskScore in the validation set of the KM survival curve distribution

In order to determine the robustness of the model, the same coefficients as obtained with the training set for the internal validation set and all data sets of TCGA were used. With this model, The RiskScore distribution of in TCGA validation set of are shown in Fig. 5a.It showed that the expression of 5 genes (*AK4, HK2, P4HA1, TGFBI* and *VEGFA*) increases as the risk value increases, which were consistent with those of the TCGA training set. The ROC analysis of RiskScore classification showed that the AUC was greater than 0.7 at 1, 3 and 5 years (Fig. 5b). Based on the calculated median of the RiskScore value, the samples were divided into either a high or low risk group and survival curves were plotted for the two groups. The two groups show a statistically significant difference with regard to prognosis, that is, the established prognosis model can significantly distinguish between risks of the different groups in the test set (Fig. 5c).

RiskScore distribution of all TCGA dataset are shown in Additional file 7: Fig. S3a. We found that *AK4, HK2, P4HA1, TGFBI* and *VEGFA* were associated with high gene expressions and a high risk related factor, results which were consistent with TCGA training set performance findings. The RiskScore classification efficiency of prognosis prediction for 1, 3, and 5 years was analyzed (Additional file 7: Fig. S3b). Based on the calculated median of RiskScore, samples were divided into high and low risk groups and a statistically significantly prognostic difference was present between two groups (Additional file 7: Fig. S3c).

Similarly, for the external validation dataset, GSE44001, the same coefficient as used for the gene in the training set model was used to calculate the RiskScore of each sample,the RiskScore grouping is presented in Additional file 8: Fig. S4a. The R software package, pROC, was used to perform the ROC analysis of riskScore prognostic classification for 1, 3 and 5 years (Additional file 8: Fig. S4b). As described above, samples were divided into high and low risk groups and survival curves were drawn. A statistically significant difference in survival was obtained between the two groups as shown in Additional file 8: Fig. S4c. These findings provide further evidence indicating that the model can not only be applied to internal, but also external data sets.

### Prognostic analysis of risk models and clinical features

**Fig. 6:**
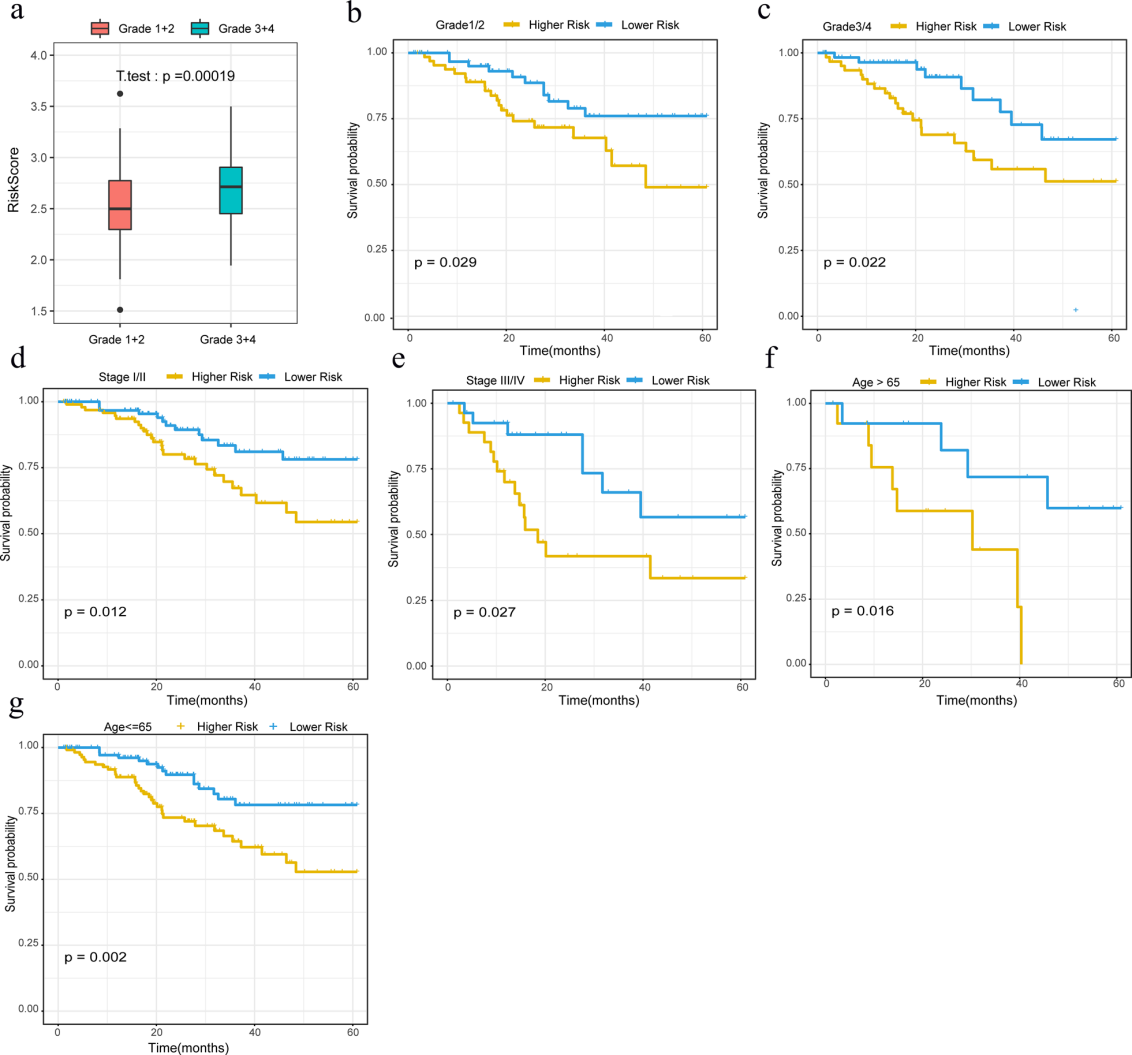
**a** Boxplot for Grade 1/2 and Grade 3/4 risk score. **b-g** Survival curves of clinical subtypes as based on Riskscore

We further performed correlation analysis with the 5-gene signature and clinical factors, it found that the box diagram, as presented in Fig. 6a, displays the distribution of RiskScore in Grade 3/4 and Grade 1/2 samples, and indicates that the Risk score of Grade 3/4 is significantly greater than that of the Grade 1/2 Risk score. The clinical subtypes, including Grade (1/2), Grade (3/4), Stages (I/II), Stages (III/IV), Age (> 65) and Age (≤ 65) were grouped based on the xpressions of Risk score, and differences in prognosis between high and low risk groups were assessed. With this analysis, low risk scores were significantly associated with a good prognosis (Fig. 6b–g).

### Enrichment analysis based on RiskScore

The KEGG and GO (BP, MF) (BP: Biological process. MF: Molecular function) items enriched within the high and low risk groups, as based on RiskScore, are shown as barplots. The specific enrichment analysis results are shown in Additional file 9: S5.csv with the KEGG pathway, including the PI3K-Akt signaling pathway, MAPK signaling pathway, Focal adhesion, TGF-beta signaling pathway and ECM-receptor interaction (Additional file 10: Fig. S5a–e).

### Univariate and multivariate cox analysis and construction of the nomogram

**Fig. 7:**
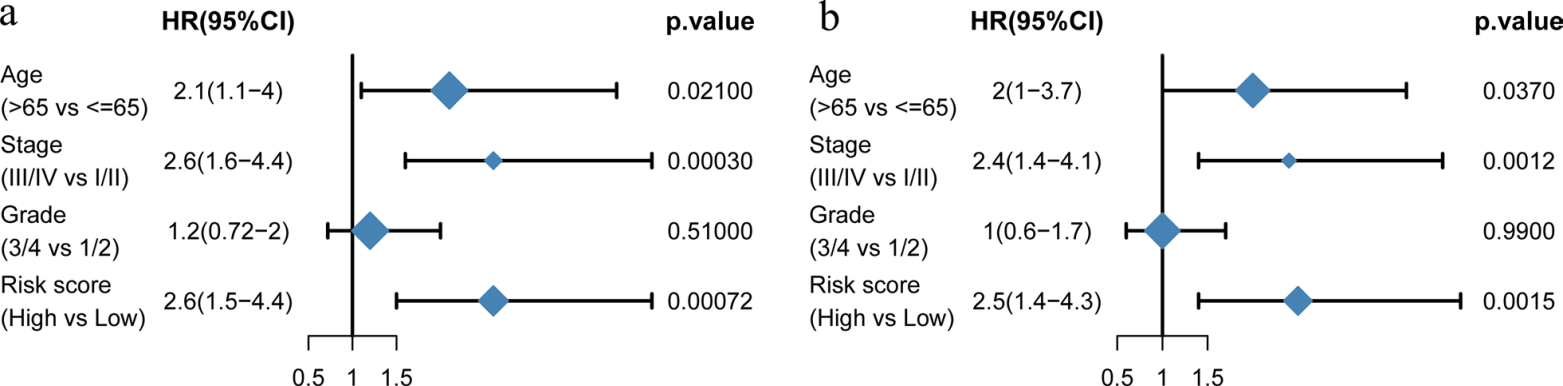
Results of clinical characteristics and RiskScore. **a** Univariate cox analysis. **b** Multivariate cox analysis

The univariate, multivariate analyses and a nomogram model was constructed to evaluate the prediction accuracy of the model. Univariate and multivariate COX regressions were used to analyze the relationship between RiskScores and other variables and clinical prognosis of patients as summarized in Fig. 7. Results from both univariate and multivariate COX analysis revealed that Age (HR > 1, p < 0.05), Stage (HR > 1, p < 0.01) and Risk Score (HR > 1, p < 0.05) were correlated with prognosis of patients. Accordingly, these factors could be used as independent risk factors for the prognosis of patients with cervical cancer.

**Fig. 8:**
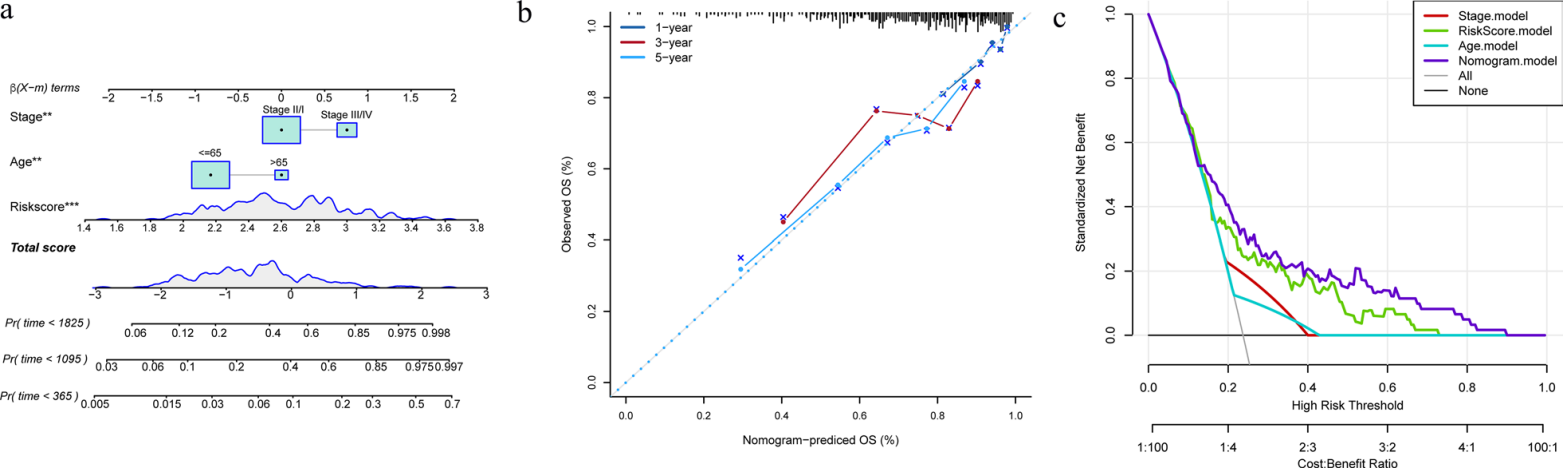
**a** Nomogram model for Stage, Riskscore and Age combinations. **b** 1, 3 and 5-year calibration curves of the Nomogram. **c** DCA of the Nomogram model

Based on the results obtained from the univariate and multivariate cox analysis, the age, Stage and RiskScore of clinical features were combined to construct a nomogram model (Fig. 8a). From this model, RiskScore features were found to exert the greatest impact on survival prediction rates, indicating that this risk model, as based on these 5 genes, can better predict the prognosis of cervical cancer. Further, a calibration curve was used to evaluate the prediction accuracy of the model, as shown in Fig. 8b. It can be seen that the predicted calibration curves of the three calibration points at 1, 3 and 5 years have a high degree of coincidence with the standard curves, indicating that the model demonstrates a good level of prediction performance. In addition, we also used DCA to evaluate the reliability of the model, as shown in Fig. 8c. This analysis reveals that the benefits of RiskScore and Nomogram are significantly greater than those of the extreme curve, where the Nomogram is greater than RiskScore and T Stage is close to the extreme curve. Such findings indicate that RiskScore and Nomogram possess a good degree of reliability. The above results demonstrate that a line graph with multiple clinical variables can better predict OS than single indicator.

### The *AK4, HK2, P4HA1, TGFBI *and *VEGFA* expression are up-regulated in cervical cancer tissue

**Fig. 9:**
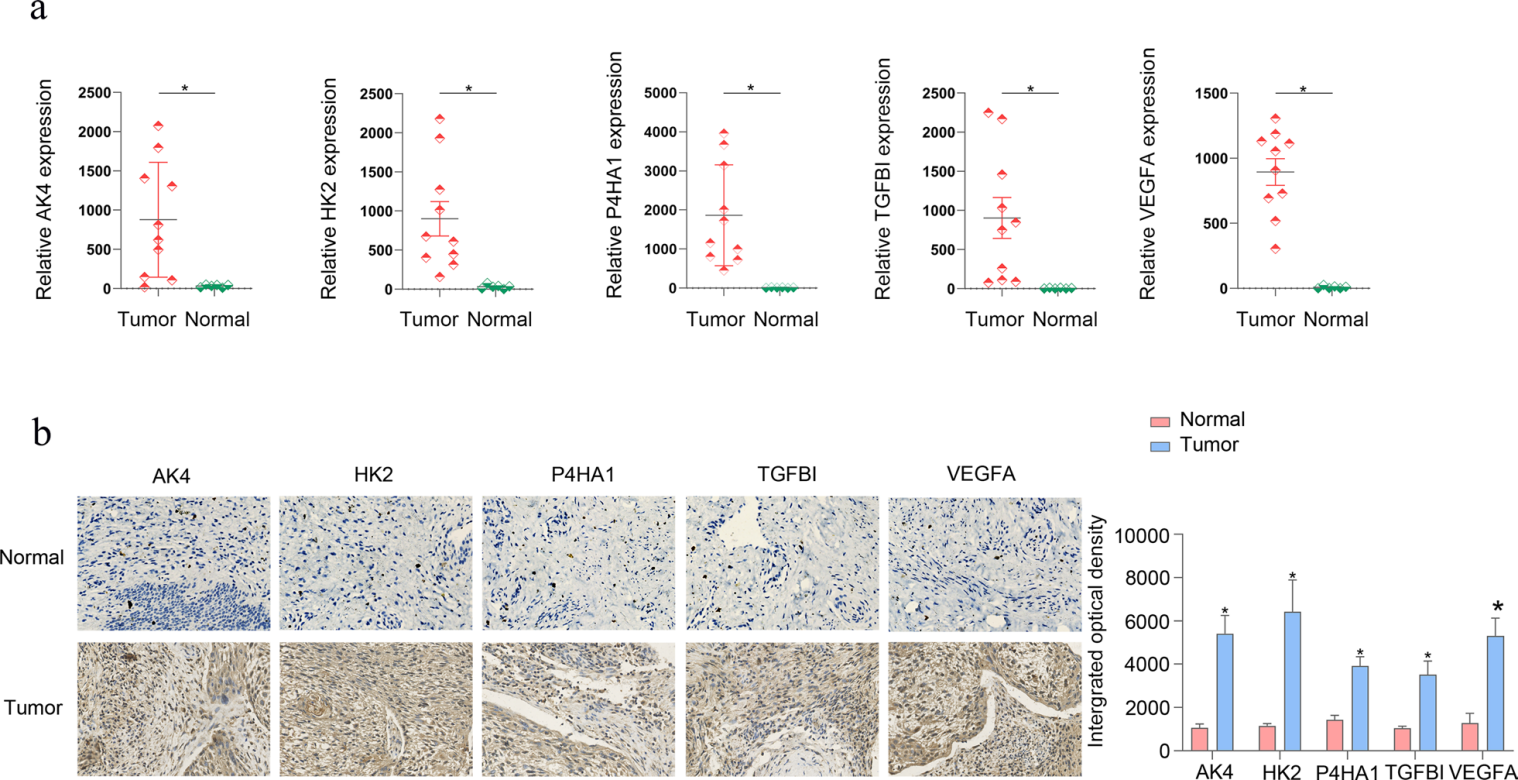
Levels of AK4, HK2, P4HA1, TGFBI and VEGFA were highly expressed in samples from cervical cancer patients. **a** RT-PCR. **b** Immunohistochemical assays. Image-ProPlus 6.0 Software was used to analyze protein expressions (*p < 0.05). Magnification: 10*20

Furthermore, to verify the accuracy of the 5-gene signature, we examined the expression of the signature genes (*AK4, HK2, P4HA1, TGFBI* and *VEGFA*) in clinical samples from 10 cervical cancer patients by qPCR and IHC analysis. The results obtained with RT-PCR (Fig. 9a) and immunohistochemistry (Fig. 0.9b) assays both showed that *AK4, HK2, P4HA1, TGFBI* and *VEGFA* were all highly expressed in these cervical cancer tissue samples. Clinical details of these 10 patients are contained in Additional file 12: S7.xls.

### The biological function of signature genes in cervical cancer cells

**Fig. 10:**
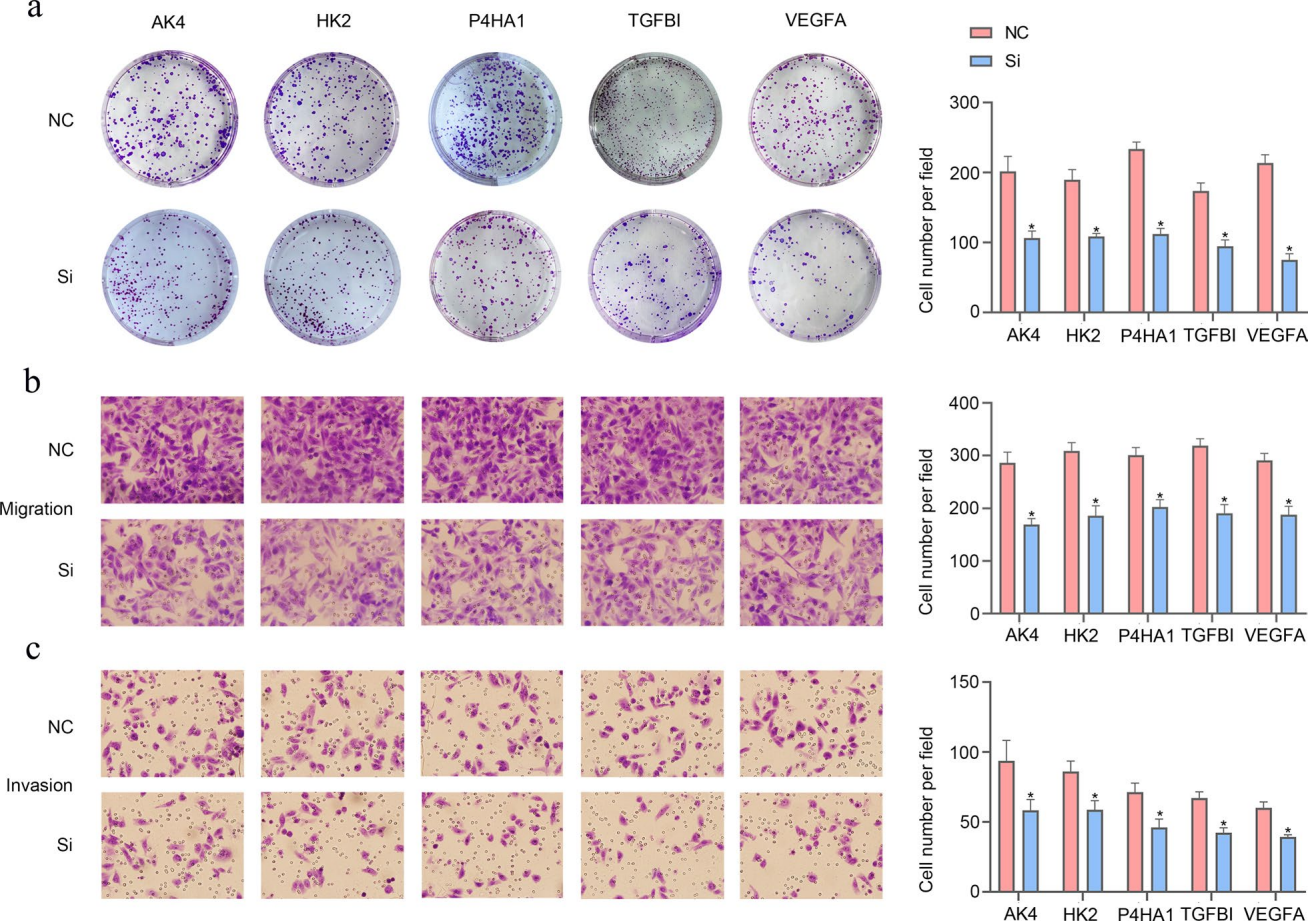
Expressions of AK4, HK2, P4HA1, TGFBI and VEGFA genes of Hela cells were silenced using RNA interference technology. Proliferation, migration and invasion of cervical cancer cells were significantly reduced in the si-AK4, si-HK2, si-P4HA1, si-TGFBI and si-VEGFA group. **a** Colony formation assay. **b** Migration. **c** Invasion. (*p < 0.05). Magnification: 10*40

To investigate the biological function of AK4, HK2, P4HA1, TGFBI and VEGFA in cervical cancer cells, transwell and colony formation assays were performed. The transwell assays were used to determine the invasion and migration ability of cervical cancer cells, and the colony formation assay was performed to detect the proliferation ability of cells. The colony formation and transwell assays results showed that reduced *AK4, HK2, P4HA1, TGFBI* and *VEGFA* expression, which significantly inhibited proliferation (Fig. 10a), migration (Fig. 10b) and invasion (Fig. 10c) ability of cervical cancer cells.

## Discussion

Cervical cancer is characterized as having a high incidence and mortality rate, particularly in low and middle economic populations of patients. Therefore, it is urgent to determine new prognostic indicators in order to more accurately predict the prognosis of patients with cervical cancer. While there have been many studies directed toward examining the relationship between hypoxia and tumor formation, the relationship between hypoxia and prognosis of patients with cervical cancer remain quite limited. In this study, the hypoxia-related gene signatures was used to predict the prognosis of patients with cervical cancer and demonstrate that this approach achieves good prediction results.

Specifically, we developed a 5-gene signature prognostic risk model and provide a verification of its validity as demonstrated in both train and test data sets. Based on the results of survival analysis, ROC and nomogram, we believe that this constructed risk factor model is quite robust. In addition, in each clinical subtype of TCGA, this risk factor model effectively predicted the risk of cervical cancer. For the first time, we have established a prognostic prediction model based on hypoxia genes. Moreover, the results of qPCR and immunohistochemistry staining assay revealed that the expression of *AK4, HK2, P4HA1, TGFBI and VEGFA* is high in cervical cancer. The functional study shown that expression of *AK4, HK2, P4HA1, TGFBI* and *VEGFA* can regulate the proliferation, migration, and invasion ability of cervical cancer cells. Among them, *AK4, P4HA1* and *TGFBI* were first confirmed as oncogene in cervical cancer. In our study, taken together, we believe that this model can be used to evaluate the prognostic risk in cervical cancer patients. And our model is better than other cervical cancer models [23, 24].

In our analysis, the high expression and high risk of these five hypoxia-related genes, *AK4, HK2, P4HA1, TGFBI* and *VEGFA*, were identified as risk factors. Adenylate kinase 4 (*AK4*) is a member of the adenosine kinase family and has been found to play an important role in malignant tumors and anti-tumor therapy. Results from recent studies have shown that high expressions of *AK4* promote lung cancer metastasis by enhancing HIF-1α stability and EMT under conditions of hypoxia [25]. High expressions of *AK4* also promote cell proliferation and invasion in ovarian cancer and *HER2* positive breast and esophageal cancers [26–28]. Hexokinase 2 (*HK2*) is a rate-limiting enzyme in the glycolysis pathway. In addition to the catalytic activity of this enzyme, *HK2* can also antagonize apoptosis within the mitochondrial pathway, which plays an important role in the invasion and metastasis of malignant tumors. It has been reported that B7-H3 promotes aerobic glycolysis and increases chemoresistance in colorectal cancer cells through the upregulation of *HK2* [29]. Silencing *HK2* in human hepatocellular carcinoma cells inhibits tumorigenesis and increases cell death, and *HK2* silencing can synergistically inhibit tumor growth with sorafenib [30]. In colorectal cancer, *PLK3* inhibits glucose metabolism by targeting HSP90/STAT3/HK2 signal transduction. Under conditions of *PLK3* overexpression, expression levels of *HK2* decrease, while silencing *PLK3* increases the expression of *HK2* in tumor cells. *HK2* silencing can inhibit the growth of colorectal cancer cells [31]. Proline 4-hydroxylase subunit α-1 (*P4HA1*) is associated with a variety of malignant tumor development pathways, such as EMT, angiogenesis, invasion, inflammation, tumor metabolism and glycolysis pathways [32]. Findings from recent studies have shown that *P4HA1* is up-regulated in lung, breast and head/neck cancer tissues, and high expression levels of *P4HA1* are significantly correlated with the clinical characteristics of these cancers. The clinical prognosis of patients with high expressions of *P4HA1* is poor [33]. In melanoma, depletion of *P4HA1* reduces cell adhesion, invasion and in vitro survival, and in xenotransplantation models, knockdown of *P4HA1* reduces the invasion of melanoma in vivo and the deposition of collagen in interstitial ECM and tumor vascular basement membranes. Such results indicate that *P4HA1* can serve as a potential biomarker for poor prognosis of primary melanoma [34]. Upregulation of *P4HA1* promotes cell migration and invasion in glioblastoma and head/neck squamous cell carcinoma and, in this way, provides a biomarker for poor prognosis in patients with high expressions of *P4HA1* [35, 36]. As an extracellular matrix protein, *TGFBI* is closely related to the development of different malignant tumors. In glioma, the median survival time for patients with a high expression of *TGFBI* was significantly shorter than that of patients showing low expressions of *TGFBI* [37]. An up-regulation of *TGFBI* can promote the occurrence and metastasis of breast cancer, increase tumor angiogenesis and increase hypoxia, and *TGFBI* overexpression promotes oral squamous cell carcinoma. Knockout of *TGFBI* inhibits the proliferation and metastasis of oral squamous cell carcinoma in vivo. Accordingly, low levels of *TGFBI* expression can predict a better prognosis [38], while patients with *TGFBI* overexpression have a poor prognosis [39]. Vascular endothelial growth factor A (*VEGFA*) plays an important role in tumor angiogenesis. *VEGFA* may be a prognostic gene in clear cell renal cell carcinoma, as significant increases in *VEGFA* are associated with a poor prognosis [40]. Upregulation of *VEGFA* also indicates a poor prognosis for lung adenocarcinoma and oral squamous cell carcinoma, suggesting that *VEGFA* represents a valuable prognostic biomarker [41, 42].

The new and important findings of this study are the identification of a prognostic 5-gene signature with a relatively high AUC in the training and test dataset, which can then predict 1, 3 and 5-year survival rates. We also established a series of clinical variables of the nomogram model and verified these predicted genes in a series of experiments, which then substantiated the reliability of the prediction. In the future, genetic diagnosis and treatment will become more effective means. The expression of 5-gene signature in cervical cancer tissues will be detected by qPCR, and the model genes will be converted into risk scores for predicting the prognosis of patients, which is significance of clinical application. The limitation of this study is that some data lacked clinical follow-up information and further genetic and experimental studies along with experimental verification will be required with larger samples. Moreover, direct clinical application tests of this prognosis model will need to be conducted.

In summary, our study developed a 5-gene signature prognostic hierarchical system based on the hypoxic pathway of cervical cancer. This protocol shows an effective AUC in the training and independent test set, and serves as a model which is independent of clinical characteristics. Further, in order to verify the signature, TCGA and GSE44001 datasets was used to test, and finally got a good risk prediction effect in those datasets. We also conducted experimental verifications on these five genes and found that the expression of *AK4, HK2, P4HA1, TGFBI* and *VEGFA* were high in cervical cancer tissues. Moreover, silencing these genes inhibited the proliferation, migration and invasion ability of cervical cancer cells as demonstrated in vitro. Therefore, these results suggesting that the signature could potentially be used to evaluate the prognostic risk of cervical cancer patients, and provide potential targets for the treatment of cervical cancer patients.

## Supplementary Information

**Additional file 1.** The cox analysis results of all hypoxia pathway-related genes.

**Additional file 2: Fig. S1.** The Cluster2 vs Cluster1 differential analysis. **a** gene volcano map. **b** gene heat map.

**Additional file 3.** Detailed differentially expressed genes.

**Additional file 4: Fig. S2.** Annotations (maps) of differentially expressed genes between Cluster2 and Cluster1. **a** KEGG. **b** BP. **c** MF.

**Additional file 5.** Detailed analysis results of KEGG pathway.

**Additional file 6.** Detailed analysis results of GO functional enrichment analyses.

**Additional file 7: Fig. S3.** **a** Expressions and clinical features of five prognostic genes in the high and low risk groups with regard to TCGA. **b** ROC curves and AUC of RiskScore classifications. **c** KM survival curve distribution of RiskScore in all TCGA sets.

**Additional file 8: Fig. S4.** **a** Expression heat maps of five model genes in the high and low risk groups with regard to GSE44001. **b** ROC curves and AUC of RiskScore classifications. **c** KM survival curve distribution of RiskScore in GSE44001.

**Additional file 9.** Specific results of enrichment analysis based on RiskScore.

**Additional file 10: Fig. S5.** **a-e** KEGG enrichment analysis results for the high and low risk groups.

**Additional file 11.** Sequences of upstream and downstream primers.

**Additional file 12.** Clinical details of these cervical cancer 10 patients.

## Data Availability

The datasets used and/or analyzed during the current study are available from the corresponding author on reasonable request.

## Abbreviations

TCGA: The Cancer Genome Atlas
HPV: Human papilloma virus
CT: Computer Tomography
MRI: Magnetic Resonance Imaging
PET-CT: Positron emission tomography computer Tomography
EMT: Epithelial-mesenchymal transition
GLUTs: Glucose transporters
GEO: Gene Expression Omnibus
OS: Overall survival
CDF: Cummulative distribution function
GO: Gene Ontology
KEGG: Kyoto Encyclopedia of Genes and Genomes
ROC: Receiver operating characteristic
DCA: Decision Curve Analysis
IHC: Immunohistochemistry
DEGs: Differential expressed genes
AUC: Area Under the Curve
BP: Biological process
MF: Molecular function
