# Supplementary figures and images for: Constructe a novel 5 hypoxia genes signature for cervical cancer
Source: Cancer Cell Int. 2021 Jul 3;21:345. doi: 10.1186/s12935-021-02050-3 (PMC8254931; doi:10.1186/s12935-021-02050-3)

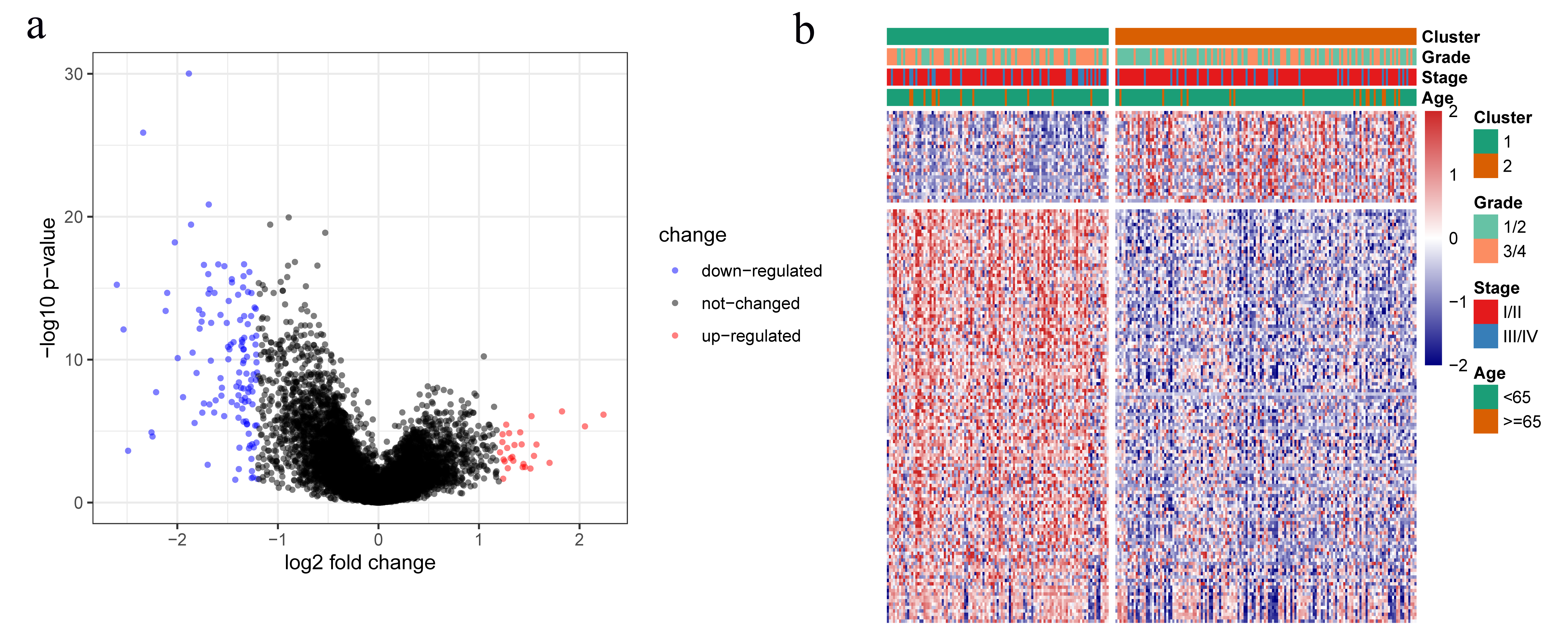

Supplement: Supplementary file 2 — Additional file 2: Fig. S1. The Cluster2 vs Cluster1 differential analysis. a gene volcano map. b gene heat map. [file 12935_2021_2050_MOESM2_ESM.png]

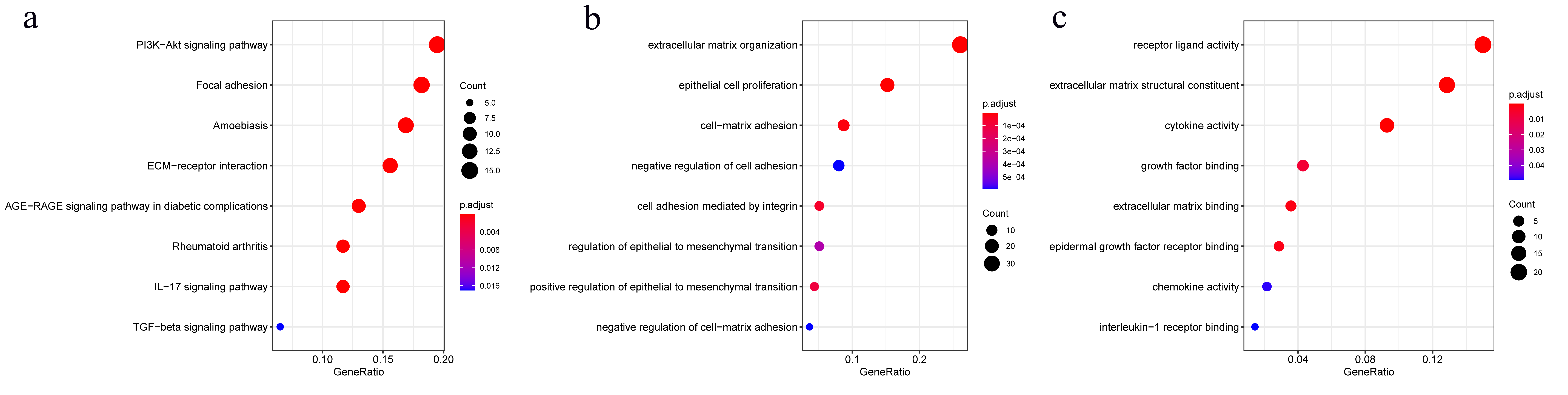

Supplement: Supplementary file 4 — Additional file 4: Fig. S2. Annotations (maps) of differentially expressed genes between Cluster2 and Cluster1. a KEGG. b BP. c MF. [file 12935_2021_2050_MOESM4_ESM.tif]

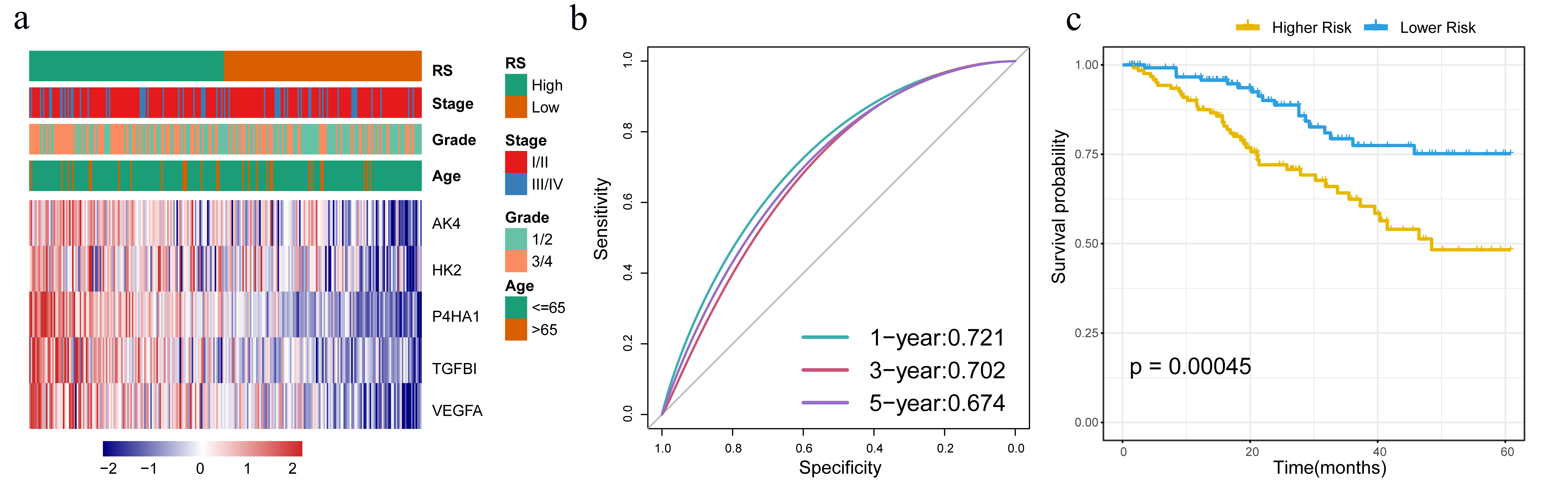

Supplement: Supplementary file 7 — Additional file 7: Fig. S3. a Expressions and clinical features of five prognostic genes in the high and low risk groups with regard to TCGA. b ROC curves and AUC of RiskScore classifications. c KM survival curve distribution of RiskScore in all TCGA sets. [file 12935_2021_2050_MOESM7_ESM.tif]

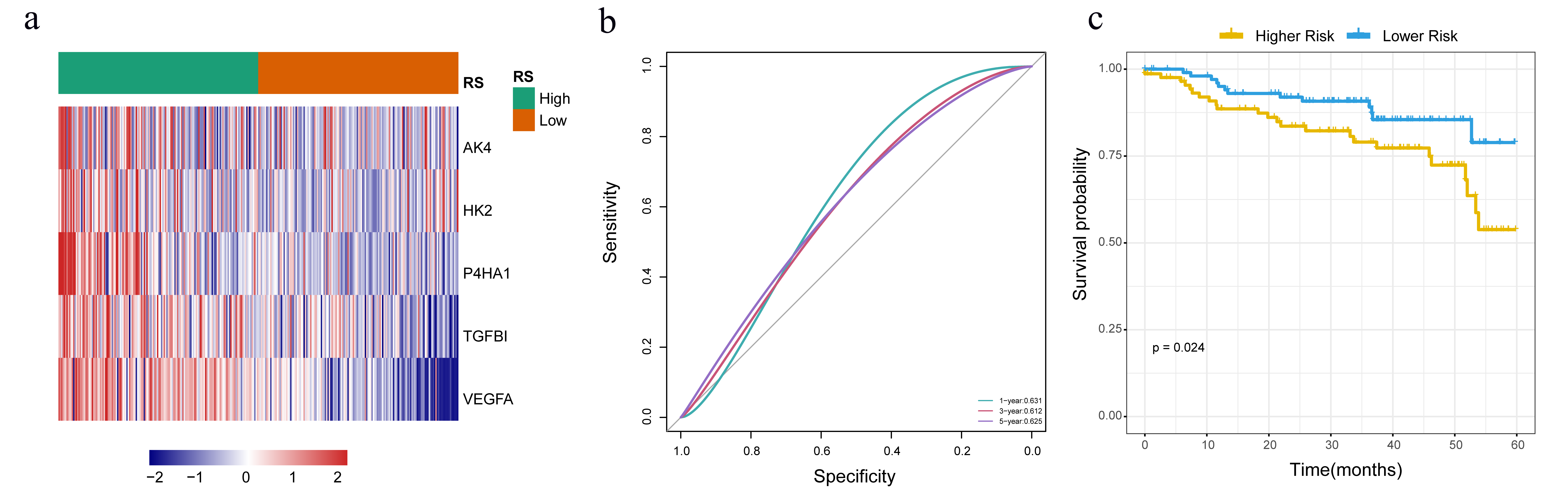

Supplement: Supplementary file 8 — Additional file 8: Fig. S4. a Expression heat maps of five model genes in the high and low risk groups with regard to GSE44001. b ROC curves and AUC of RiskScore classifications. c KM survival curve distribution of RiskScore in GSE44001. [file 12935_2021_2050_MOESM8_ESM.png]

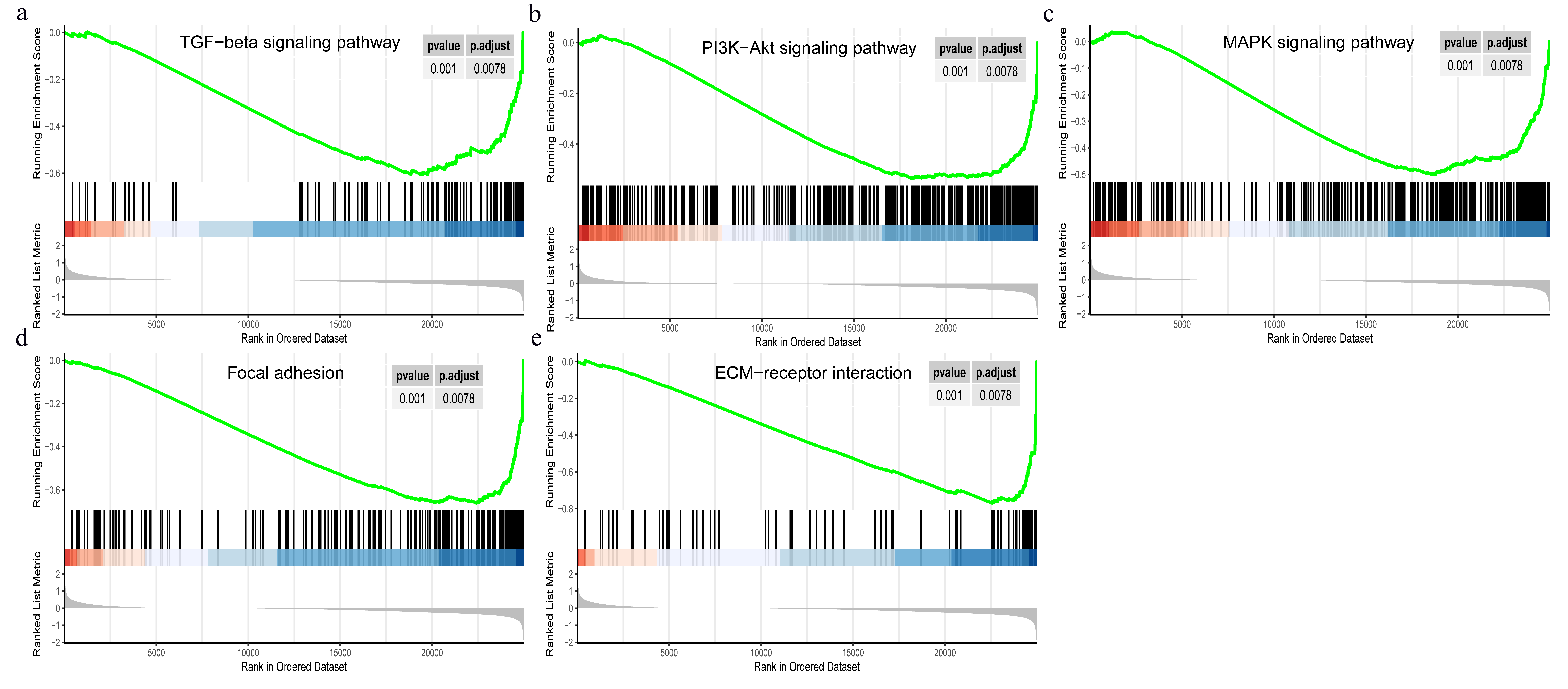

Supplement: Supplementary file 10 — Additional file 10: Fig. S5. a-e KEGG enrichment analysis results for the high and low risk groups. [file 12935_2021_2050_MOESM10_ESM.tif]
